# 

**Published:** 1934

**Authors:** 


					(I . ? '
Vol. LI. No. 194.
I
I m
' Jfr'
WINTER, 1934.
ml
The Bri
I Medico-Chirurgi
',?.me?\ ..   ,.^J
PUBLISHED trtrilBB THE AUSPICES OF
THE BRISTOL MEDICO-CHIRURGICAL SOCIETY.
f Editor : J. A. NIXON, C.M.G., M.D., E.R.C.P.
.? .-?? <
WITH WHOM ABE ASSOCIATED
' ' - y ? ?
E. W. HEY GROVES, D.Sc., IIS., F.R.C.S.
A. E. ILES, O.B.E., M.B., B.S., F.R.C.S.
A. RENDLE SHORT, M.D., B.S., B.Sc>, F.R.C.S.
E. WATSON-WILLIAMS, M.C., Ch.M., P.R.O.S., Assistant Editor.
Editorial Secretary : A. L. FLEMMING, M.B., Oh.B.
I .
BRISTOL: J. W. ABROWSMTTH LTI)!, 11 QUAY S^ftEET.
London : J. W. Abjbowsmeth (London) Ltd., 8 ENDauEMHGIbd??, yf.d. 1.
^ Price Three Shillings ne^
1 V
iTGRJ1^
I "vir 4 1 ? r"l ?' ' sS
V . ? ' *\ *
(jff* < '

				

